# Role of Programmed Cell Death in the Pathogenesis of Kawasaki Disease: Mechanisms and Therapeutic Implications

**DOI:** 10.1155/mi/7913718

**Published:** 2026-05-06

**Authors:** Bo Ding, Ziyu Huang, Ailixiati Alifu, Kaijing Wang, Dufei Zhang, Yazhou Wang, Renwei Chen

**Affiliations:** ^1^ School of Pediatrics, Hainan Medical University, Haikou, 571199, Hainan Province, China, hainmc.edu.cn; ^2^ Department of Cardiothoracic Surgery, Hainan Women and Children’s Medical Center, Haikou, 570300, Hainan Province, China; ^3^ Department of Pediatric Vasculocardiology, Hainan Women and Children’s Medical Center, Haikou, 570300, Hainan Province, China

**Keywords:** apoptosis, Kawasaki disease, molecular mechanism, programmed cell death

## Abstract

Programmed cell death (PCD) is a genetically regulated, orderly cell death process essential for tissue homeostasis. Contemporary PCD comprises a broad spectrum, including apoptosis, pyroptosis, ferroptosis, necroptosis, autophagy‐dependent cell death, NETosis, parthanatos, and entotic cell death. Kawasaki disease (KD) is an acute pediatric systemic vasculitis and a leading cause of acquired childhood heart disease, with coronary artery lesions as the most severe complication. Mounting evidence confirms that PCD is tightly associated with KD‐related vascular endothelial injury and inflammatory amplification. Among all PCD subtypes, apoptosis, pyroptosis, and ferroptosis have the most sufficient and direct evidence in KD pathogenesis, mediating vascular wall damage and inflammatory imbalance via distinct molecular pathways. This review focuses on these three well‐documented PCD forms (with a brief overview of other PCD subtypes and their potential KD relevance), systematically elaborates their crosstalk with KD pathogenesis, summarizes current research progress, and proposes targeted therapeutic strategies for KD.

## 1. Introduction

Kawasaki disease (KD) is an acute, self‐limiting febrile disorder in children that primarily affects medium‐sized arteries, with the coronary arteries being most significantly involved. It is one of the leading causes of acquired heart disease in children in developed countries, ranking second only to IgA vasculitis in incidence [1, 2]. Typical clinical presentation includes persistent fever accompanied by bilateral nonsuppurative conjunctivitis, diffuse oral mucosal inflammation, polymorphic rash, firm edema of the hands and feet, and nonsuppurative cervical lymphadenopathy. Although most children (75%–85%) recover spontaneously, some develop coronary artery lesions, ranging from asymptomatic mild dilation to aneurysm formation. Giant coronary artery aneurysms are prone to secondary thrombosis, myocardial infarction, and sudden death [2]. Currently, the mainstay of treatment is intravenous immunoglobulin (IVIG) combined with aspirin; however, some patients show treatment resistance or experience disease recurrence. Emerging evidence suggests that vasculitis in KD is closely associated with inflammatory factor‐mediated cell death within the vascular wall [3]. Programmed cell death (PCD) is a highly conserved gene‐regulated cell death mode. In addition to the well‐studied apoptosis, pyroptosis, and ferroptosis, the contemporary PCD family also includes necroptosis (RIPK1/RIPK3/MLKL‐dependent), autophagy‐dependent cell death, NETosis (neutrophil extracellular trap‐mediated cell death, highly relevant to vasculitis), parthanatos (PARP‐1‐dependent), and entotic cell death [4]. Among these subtypes, apoptosis, pyroptosis, and ferroptosis have been verified to participate in KD endothelial injury and inflammatory responses by direct clinical, animal, and cell experiments; other PCD forms are still in the preliminary exploration stage in KD [5]. This review focuses on the three core PCD subtypes with clear KD relevance, supplemented by the potential role of necroptosis in KD vascular inflammation, to systematically clarify the molecular mechanism of PCD in KD and provide new targets for clinical treatment [6].

## 2. Pathogenesis of KD

### 2.1. Immune System Abnormal Activation

**Figure 1:**
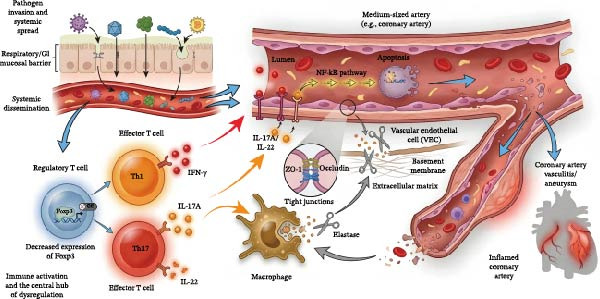
Overview of immune system dysregulation in patients with Kawasaki disease.

KD is induced by pathogen infection in genetically susceptible individuals, leading to systemic immune overactivation (Figure 1). Pathogens (EB virus, coxsackievirus, SARS‐CoV‐2, *Streptococcus pyogenes*, etc.) invade the body, activate T cells, macrophages, and other immune cells, trigger an immune cascade, and release a large number of inflammatory mediators, causing systemic vascular inflammation. Imbalanced T‐cell activation is a key factor in KD severity [7]. Foxp3, a core regulatory molecule of Treg cells, is significantly downregulated in KD patients, leading to excessive activation of effector T cells [8]. Th1 cells secrete IFN‐γ to activate endothelial NF‐κB and induce apoptosis; Th17 cells secrete IL‐17A/IL‐22 to destroy endothelial tight junctions; activated macrophages release elastase to degrade vascular basement membrane, jointly causing vascular endothelial injury.

### 2.2. Release of Inflammatory Cytokines

Acute KD is accompanied by a sharp increase in proinflammatory cytokines. TNF‐α directly damages vascular endothelial cells (VECs) and is closely related to coronary aneurysm formation. IL‐1, IL‐18, IL‐6, and other cytokines synergistically activate NF‐κB and other pathways, further amplifying inflammation and endothelial damage. TNF‐α‐targeted therapy has shown protective effects on KD‐induced endothelial injury in animal models [9].

### 2.3. Vascular Endothelial Injury and Dysfunction

Damage and dysfunction of VECs represent the initiating events of vasculitis. When endothelial cells are stimulated by pathogens or immune complexes, surface adhesion molecules are markedly upregulated, facilitating the adhesion and transmigration of inflammatory cells, such as monocytes and neutrophils, into the vascular intima [10]. This process not only activates the complement system but also aggravates endothelial injury through the release of reactive oxygen species (ROS) and proteases.

**Figure 2:**
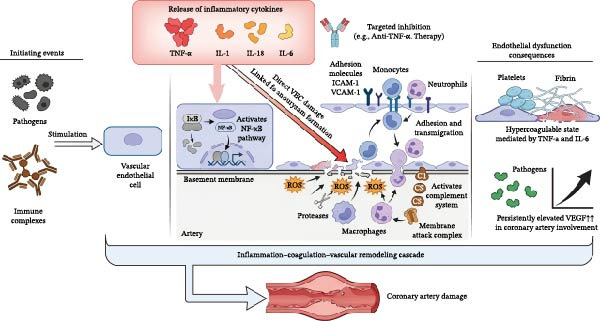
Overview of inflammatory cytokine release and vascular endothelial damage in patients with Kawasaki disease.

Children with KD in the acute phase often exhibit a hypercoagulable state, which is closely associated with endothelial dysfunction mediated by inflammatory cytokines, including TNF‐α and IL‐6 [11]. Vascular endothelial growth factor, a key regulator of angiogenesis and vascular permeability, remains persistently elevated in patients with KD, particularly in those with coronary artery involvement, in whom levels are significantly higher than those in patients without coronary complications [12]. Collectively, these findings indicate that endothelial dysfunction driven by an inflammatory–coagulation–vascular remodeling cascade is a central mechanism underlying coronary artery damage in KD (Figure 2).

### 2.4. Regulation of Endothelial Progenitor Cells (EPCs)

EPCs, which serve as precursors for endothelial cells, play a pivotal role in angiogenesis and the repair of endothelial injury. A previous study [13] reported that patients with acute KD exhibited significantly elevated EPC counts in peripheral blood, suggesting compensatory mobilization to facilitate endothelial repair. However, during this phase, EPCs show markedly impaired proliferation, adhesion, and migration capacities, with the severity of functional dysfunction inversely correlated with serum levels of inflammatory markers, such as TNF‐α and C‐reactive protein. Although the mechanisms underlying stem cell‐based therapies in KD are still under investigation, their clinical efficacy requires further validation through multicenter randomized controlled trials.

## 3. Molecular Mechanism of PCD

### 3.1. Cell Apoptosis

Apoptosis, a key mechanism of PCD, plays a central role in physiological processes such as embryonic development, maintenance of tissue homeostasis, and clearance of inflammatory cells by precisely regulating the balance between cell proliferation and death. Apoptotic execution follows a highly ordered molecular program divided into three interconnected pathways: the extrinsic (death receptor‐mediated) pathway, the intrinsic (mitochondrial) pathway, and the ER stress‐mediated pathway [14].

#### 3.1.1. Extrinsic Apoptosis Pathway

The extrinsic pathway is triggered by extracellular death ligands binding to cell‐surface death receptors, including TNF‐α/TNFR1, FasL/Fas, and TRAIL/DR4/DR5. Upon TNF‐α binding, TNFR1 recruits TRADD, TRAF2, RIPK1, and cIAPs to form membrane‐associated Complex I, which primarily mediates prosurvival and proinflammatory signaling via NF‐κB activation without inducing apoptosis [15]. Following receptor internalization and signaling remodeling, TRADD dissociates from Complex I and assembles with FADD and procaspase‐8 to form cytosolic Complex II, inducing the autocatalytic activation of caspase‐8 [16]. Active caspase‐8 subsequently cleaves and activates effector caspases (caspase‐3, ‐6, and ‐7), which degrade cellular substrates, leading to apoptotic cell death without inflammatory leakage.

#### 3.1.2. Intrinsic (Mitochondrial) Apoptosis Pathway

The intrinsic pathway is activated by intracellular stress signals, including DNA damage, oxidative stress, and nutrient deprivation. BH3‐only proteins (Bim, Bid, Bad, and Puma) are activated and antagonize antiapoptotic Bcl‐2/Bcl‐xL, thereby releasing proapoptotic BAX/BAK [17]. BAX/BAK oligomerization increases mitochondrial outer membrane permeability (MOMP), releasing cytochrome c, Smac/DIABLO, and Omi/HtrA2 into the cytosol. Cytochrome c subsequently binds Apaf‐1 and procaspase‐9 to form the apoptosome, activating caspase‐9, which in turn activates effector caspases to culminate in apoptosis [18].

#### 3.1.3. ER Stress‐Mediated Apoptosis

The ER is a central organelle for protein folding and calcium homeostasis, and disruption of its function triggers the unfolded protein response (UPR). Chronic or severe ER stress activates apoptotic signaling through two key mechanisms: the PERK–eIF2α–ATF4–CHOP axis, in which sustained PERK activation—as demonstrated in vascular cells exposed to inflammatory stimuli such as TNF‐α—drives CHOP induction, thereby suppressing antiapoptotic Bcl‐2 and activating proapoptotic effectors [19]; and activation of ER‐resident caspase‐4 (the human functional orthologue of murine caspase‐12), which directly triggers caspase‐3 [20]. Both mechanisms converge on the execution of apoptosis and are particularly relevant to conditions characterized by vascular inflammatory injury and oxidative stress.

#### 3.1.4. Apoptosis in KD

Apoptotic signaling has been implicated in the endothelial injury characteristic of KD. An enzyme‐linked immunosorbent assay of patient serum has demonstrated significant downregulation of SIGIRR alongside elevated caspase‐8 levels compared with healthy controls, while SIGIRR expression was similarly reduced in KD‐treated endothelial cells in vitro. These findings suggest that the loss of SIGIRR‐mediated suppression of TLR4 signaling may lower the apoptotic threshold in VECs. Consistent with this, overexpression of SIGIRR was shown to alleviate endothelial apoptosis by inhibiting caspase‐8 activation, identifying the SIGIRR–caspase‐8 axis as a potential mechanistic link between innate immune dysregulation and endothelial apoptosis in KD [21].

### 3.2. Cell Pyroptosis

Pyroptosis is a proinflammatory, lytic PCD modality defined by gasdermin‐mediated membrane pore formation and the release of mature IL‐1β and IL‐18. It occurs via canonical and noncanonical pathways with extensive crosstalk.

#### 3.2.1. Canonical Pyroptosis Pathway

The canonical pathway is driven by inflammasome assembly: PRRs (NLRP3, NLRP1, and AIM2) recognize pathogen‐associated molecular patterns (PAMPs) or damage‐associated molecular patterns (DAMPs). Inflammasomes recruit and activate procaspase‐1, which undergoes autocleavage. Active caspase‐1 cleaves GSDMD to generate the N‐terminal pore‐forming domain (GSDMD‐N). GSDMD‐N inserts into the plasma membrane, forming 10–15 nm pores, causing cell swelling, osmotic lysis, and release of cytoplasmic contents. Caspase‐1 also matures pro‐IL‐1β and pro‐IL‐18 into their bioactive forms, which are secreted through GSDMD pores to amplify inflammation [22, 23].

#### 3.2.2. Noncanonical Pyroptosis Pathway

The noncanonical pathway is directly activated by intracellular lipopolysaccharide (LPS): LPS binds and activates human caspase‐4/5 or mouse caspase‐11. Active caspases cleave GSDMD to generate GSDMD‐N, inducing membrane lysis. Critical crosstalk: GSDMD pore formation induces K+ efflux, which secondarily activates the NLRP3 inflammasome and caspase‐1, leading to IL‐1β/IL‐18 maturation [23]. GSDMB modulates caspase‐4 activity and regulates GSDMD cleavage, adding an additional layer of regulatory complexity.

### 3.3. Cell Ferroptosis

Ferroptosis is an iron‐dependent, nonapoptotic, oxidative PCD modality driven by lethal lipid peroxidation. It is regulated by three core axes:

#### 3.3.1. Iron Metabolism Dysregulation

Extracellular Fe^3+^ is imported via transferrin receptor 1 (TFR1), reduced to Fe^2+^ by STEAP3, and stored in the labile iron pool (LIP). Excess Fe^2+^ promotes Fenton reactions, generating highly reactive ROS that induce lipid peroxidation [24].

#### 3.3.2. Lipid Peroxidation

Polyunsaturated fatty acids (PUFAs) are activated by ACSL4 and esterified into phosphatidylethanolamine (PE) by LPCAT3. Lipoxygenases (LOXs) catalyze the peroxidation of PUFA‐PE conjugates, generating lethal lipid peroxides [24].

#### 3.3.3. Antioxidant System Failure

Glutathione (GSH) peroxidase 4 (GPX4) is the central defender against ferroptosis, reducing lipid peroxides to nontoxic alcohols using GSH as a cofactor. GSH depletion or GPX4 inactivation leads to unchecked lipid peroxidation and ferroptotic cell death [24].

### 3.4. Cell Necroptosis

Necroptosis is a caspase‐independent, inflammatory form of regulated necrosis mediated by the RIPK1–RIPK3–MLKL signaling axis: triggered by death receptors (TNFR1), TLRs, or intracellular stress. RIPK1 is activated and recruits RIPK3 to form a necrosome. RIPK3 phosphorylates MLKL, which oligomerizes and translocates to the plasma membrane, inducing membrane permeabilization and inflammatory cell lysis [25]. Necroptosis is strongly implicated in systemic vasculitis and vascular inflammation, making it highly relevant to KD pathogenesis, although direct evidence remains limited.

## 4. Relationship Between PCD and KD

### 4.1. PDCD4, miR‐223, and VEC Apoptosis in KD

PDCD4 is a multifunctional tumor suppressor and inflammatory regulator that plays an important role in VECs. It primarily functions by inhibiting anti‐inflammatory signaling pathways, promoting the expression of proinflammatory factors and adhesion molecules, suppressing antiapoptotic proteins, activating caspase cascades to enhance apoptotic sensitivity, and inhibiting VEC proliferation and migration [26]. During the acute phase of KD, large amounts of TNF‐α are released and bind to TNFR1 on VECs. This interaction activates the NF‐κB pathway via TRADD/TRAF2 signaling, which in turn activates κB‐binding sites within the PDCD4 promoter region, inducing PDCD4 transcription and increasing both its mRNA and protein expression [26].

Zheng et al. [27] conducted in vitro experiments using serum samples collected from patients with KD during the acute febrile phase, after IVIG treatment, and from healthy controls. Compared with controls, patients with KD showed significantly increased serum expression of miR‐223‐3p, RORγt, and Th17 cells, accompanied by reduced levels of TGF‐β1, FOXP3, and regulatory T cells (Tregs). In parallel, serum concentrations of IL‐6, IL‐17, and IL‐23 were markedly elevated, whereas IL‐10 and FOXP3 levels were significantly reduced. These immunological alterations were reversed following IVIG treatment. Furthermore, KD serum increased miR‐223‐3p expression and suppressed FOXP3 expression in human coronary artery endothelial cells (HCAECs). Overexpression of miR‐223‐3p promoted apoptosis in HCAECs, an effect that was attenuated by the IVIG‐treated serum. Serum from patients with KD downregulated FOXP3, Bcl‐2, TGF‐β1, and IL‐10 expression, while upregulating caspase‐3, BAX, IL‐17, IL‐6, and IL‐23; IVIG‐treated serum produced the opposite effects. Notably, KD serum significantly increased PDCD4 expression, thereby activating caspase‐3 and caspase‐9 signaling pathways and promoting VEC apoptosis [26].

In a related study, Chu et al. [28] further explored the upstream regulatory mechanism of miR‐223 in KD‐associated vascular injury, demonstrating that bone marrow‐derived miR‐223 functions as an endocrine genetic signal that is transferred to VECs via circulation. In KD, elevated bone marrow‐derived miR‐223 was delivered to VECs, where it modulated downstream targets involved in vascular inflammation and endothelial injury. This intercellular miR‐223 signaling axis contributed to the dysregulation of endothelial homeostasis, providing a potential mechanistic link between systemic immune activation and local vascular damage in KD.

However, most of these findings were derived from in vitro experiments. Although such models can partially recapitulate key pathological features observed in vivo, inherent limitations—including their single‐cell nature, static conditions, and lack of multicellular interactions—restrict their ability to fully reflect the complex disease microenvironment. Therefore, these experimental results should be interpreted cautiously and further validated using animal models and clinical samples to avoid overinterpretation.

### 4.2. KD and Cell Pyroptosis

In a study by Si et al. [29], experiments using HCAECs from KD demonstrated that LL‐37 (cathelicidin) was highly expressed in KD. Following stimulation with LL‐37, expression of TLR4, NLRP3, and multiple inflammatory factors was markedly elevated in HCAECs. Importantly, intervention with TLR4‐specific inhibitors effectively attenuated the LL‐37‐induced upregulation of TLR4, NLRP3, and inflammatory mediators, suggesting the TLR4‐NF‐κB‐NLRP3 signaling axis as a potential therapeutic target for KD.

Jia et al. [30] investigated clinical samples from patients with KD and observed significantly elevated serum levels of pyroptosis‐related proteins, including apoptosis‐associated speck‐like protein containing a CARD (ASC), caspase‐1, IL‐1β, IL‐18, GSDMD, and lactate dehydrogenase, compared with healthy controls. Their study further demonstrated that high mobility group box 1 (HMGB1)/receptor for advanced glycation end product (RAGE)/histone protease B signaling activated NLRP3‐dependent endothelial pyroptosis. These findings indicate that endothelial pyroptosis plays a critical role in coronary artery endothelial injury in KD.

Mechanistically, ATP released from injured VECs binds to P2X7 receptors on immune cell surfaces, inducing membrane pore formation and intracellular potassium efflux. Following viral infection or immune cell activation, mitochondrial ROS accumulate, leading to oxidative damage and triggering conformational activation of NLRP3. Neutrophil‐derived tissue protease B subsequently enters the cytoplasm and directly activates the NLRP3 inflammasome. Then, activated NLRP3 associates with ASC to form functional inflammasome complexes [31]. Active caspase‐1 cleaves pro‐IL‐1β and pro‐IL‐18 into their mature forms, which bind to receptors on VECs, activate the NF‐κB signaling pathway, and induce endothelial apoptosis. In parallel, caspase‐1 cleaves GSDMD to generate the N‐terminal fragment (GSDMD‐N), which can induce paracrine pyroptosis in neighboring endothelial cells.

Pyroptosis‐driven inflammation further promotes vascular smooth muscle cell proliferation and collagen deposition, contributing to coronary artery stenosis. The combined effects of endothelial injury and inflammatory infiltration reduce vascular wall elasticity and structural integrity, ultimately leading to coronary artery dilation or aneurysm formation and exacerbating disease severity in patients with KD [29, 30].

### 4.3. KD and Ferroptosis

Zhao et al. [32] downloaded expression and validation datasets from the Gene Expression Omnibus database and used the R software package limma to identify differentially expressed RNAs between patients with KD and healthy controls. By intersecting these results with ferroptosis‐related genes (FRGs), KD‐associated FRGs were identified, followed by Gene Ontology (GO) and Kyoto Encyclopedia of Genes and Genomes enrichment analyses. The results demonstrated that genes in patients with KD were significantly enriched in Toll‐like receptor signaling pathways, NOD‐like receptor signaling pathways, ferroptosis, and necrotic apoptosis processes. Among these, IL‐1β and TIMP1 were identified as key upregulated FRGs in KD. R‐based differential expression analysis revealed significant differences between patients with KD and healthy individuals, suggesting that FRGs, such as IL‐1β and TIMP1, play important roles in the onset and progression of KD.

Further, Song et al. [33] demonstrated that KD‐induced abnormal gene expression can trigger cellular ferroptosis by promoting intracellular iron transport and lipid accumulation while inhibiting the GSH/GPX4 antioxidant signaling axis. Ferroptosis was shown to mediate key pathological processes, including KD‐associated vascular endothelial injury, oxidative stress, inflammatory responses, and immune cell infiltration. Clinically, abnormally elevated expression of markers, such as IL‐1β, TLR4, MAPK14, LCN2, and ALOX5, may serve as potential diagnostic biomarkers for KD.

## 5. Prospects for KD Treatment

To target apoptotic pathways (Table 1), therapeutic agents, such as anti‐TNF‐α monoclonal antibodies and TNF‐α receptor fusion proteins, can be used to interfere with TNF‐α/TNFR1 signaling, thereby blocking the initiation of the extrinsic apoptosis pathway. Suad et al. [34] demonstrated that the use of these agents as second‐line therapy in IVIG‐nonresponsive patients with severe KD significantly reduced serum TNF‐α levels and effectively inhibited endothelial cell apoptosis, ultimately improving patient prognosis. In addition, caspase‐3 and caspase‐8 inhibitors can be employed to suppress the execution phase of apoptosis, thereby preventing KD serum‐induced extrinsic endothelial cell apoptosis.

**Table 1:** Targeting apoptotic pathways in Kawasaki disease.

Target	Mechanism of action	Intervention method	Research evidence
TNF‐α/TNFR1	Blocking the initiation signal of the exogenous apoptosis pathway	Infliximab (anti‐TNF‐α monoclonal antibody); etanercept (TNF‐α receptor fusion protein)	Infliximab has been used in IVIG‐unresponsive severe KD to reduce serum TNF‐α levels and decrease endothelial cell apoptosis
Caspase‐3/caspase‐8	Inhibition of apoptosis execution cascade	Z‐DEVD‐FMK (caspase‐3 inhibitor); Z‐IETD‐FMK (caspase‐8 inhibitor)	Z‐IETD‐FMK can block the exogenous apoptosis of endothelial cells induced by KD serum and reduce cell shedding

Abbreviations: IVIG, intravenous immunoglobulin; KD, Kawasaki disease; TNF‐α, tumor necrosis factor‐alpha; TNFR1, TNF receptor 1.

Agents, such as colchicine and NLRP3 inhibitors, can be used to target the pyroptotic pathway (Table 2). These drugs act on the NLRP3 inflammasome signaling axis to inhibit inflammasome assembly, block the initiation of pyroptosis, and disrupt upstream inflammatory activation signals. Alternatively, caspase‐1 inhibitors can be administered to suppress caspase‐1 activity, thereby inhibiting IL‐1β maturation and GSDMD cleavage. This strategy effectively reduces endothelial cell pyroptosis and alleviates endothelial injury.

**Table 2:** Targeting the pyroptosis pathway in Kawasaki disease.

Target	Mechanism of action	Intervention method	Research evidence
NLRP3 inflammatory bodies	Inhibition of inflammasome assembly and blocking of pyroptosis initiation	Colchicine (inhibits NLRP3 oligomerization); MCC950 (specific NLRP3 inhibitor)	MCC950 reduces serum IL‐1β/IL‐18 levels and decreases coronary pyroptosis cell infiltration in KD mice
Caspase‐1	Inhibition of caspase‐1 activation and blocking of IL‐1β maturation and GSDMD cleavage	VX‐765 (oral caspase‐1 inhibitor)	VX‐765 can block serum‐induced pyroptosis of endothelial cells in patients with KD and reduce IL‐1β release

Abbreviations: IL‐18, interleukin‐18; IL‐1β, interleukin‐1beta; KD, Kawasaki disease; NLRP3, NOD‐, LRR‐, and pyrin domain‐containing protein 3.

In ferroptosis‐targeting pathways (Table 3), deferiprone counteracts the intracellular iron overload by eliminating excess free iron. This mechanism blocks the initiation of the Fenton reaction and lipid peroxidation, thereby inhibiting the accumulation of lipid peroxidation markers. Additionally, GPX4 activators can enhance the GPX4 system and boost the antioxidant capacity of the body. This approach accelerates the clearance of lipid peroxidation products, suppresses endothelial permeability, and supports the disease treatment.

**Table 3:** Targeting the ferroptosis pathway in Kawasaki disease.

Target	Mechanism of action	Intervention method	Research evidence
Intracellular iron overload	Clearing free iron and blocking Fenton reaction and initiation of lipid peroxidation	DFO (ferrochelate)	DFO reduces iron accumulation in endothelial cells and decreases lipid peroxidation markers in a model of KD mice
GPX4 system	Enhancing antioxidant capacity and removing lipid peroxidation products	Ferrostatin‐1 (Fer‐1, GPX4 activator)	Fer‐1 dose‐dependently inhibits serum‐induced ferroptosis in patients with KD, upregulates GPX4 expression, and reduces endothelial permeability

Abbreviations: Fer‐1, ferrostatin‐1; GPX4, glutathione peroxidase 4; KD, Kawasaki disease.

## 6. Limitations, Controversies, and Future Perspectives

### 6.1. Current Limitations

1. Evidence imbalance: Apoptosis and pyroptosis are well‐supported; ferroptosis remains speculative; other PCD modalities are unstudied.
2. Model limitations: Most data are from in vitro systems or LCWE mouse models, which do not fully recapitulate human KD genetics and pathology.
3. Clinical translation gap: Most PCD‐targeted agents are preclinical; few have entered KD clinical trials.
4. Crosstalk complexity: The precise regulatory network between PCD pathways in KD remains incompletely defined.
5. Biomarker gap: No validated PCD‐related biomarkers for predicting IVIG resistance or CALs.

### 6.2. Controversies

1. Primary driver pathway: It remains unclear whether apoptosis, pyroptosis, or ferroptosis is the dominant driver of endothelial injury in KD.
2. Ferroptosis causality: Whether ferroptosis is a cause or a consequence of KD vascular injury remains debated.
3. Necroptosis relevance: The role of necroptosis in KD is entirely untested.

### 6.3. Future Research Directions

1. In vivo validation: Use humanized KD models to confirm the role of ferroptosis and necroptosis in CAL formation.
2. Biomarker discovery: Identify PCD‐related signatures for early risk stratification.
3. Clinical trials: Evaluate PCD‐targeted agents (anti‐TNF and NLRP3 inhibitors) in IVIG‐resistant KD.
4. Mechanistic crosstalk: Define the spatiotemporal regulation of PCD pathways in the coronary endothelium.
5. Novel targets: Explore necroptosis, NETosis, and ER stress as therapeutic targets.

## 7. Summary

KD, a leading cause of acquired heart disease in children, arises from a complex interplay of immune dysregulation, excessive inflammatory cytokine release, vascular endothelial injury, and endothelial dysfunction. PCD is deeply involved in KD‐associated vascular damage through three principal mechanisms: apoptosis, pyroptosis, and ferroptosis. Activation of the caspase cascade mediated by PDCD4, inflammasome‐driven pyroptosis regulated by NLRP3, and ferroptosis triggered by dysregulated iron metabolism collectively exacerbate vascular inflammation and pathological remodeling, representing critical pathways in the progression of KD.

From a therapeutic perspective, several targeted strategies have emerged. Apoptotic pathways can be inhibited by anti‐TNF‐α monoclonal antibodies or TNF‐α receptor fusion proteins that block TNF‐α/TNFR1 signaling, as well as by caspase‐3 and caspase‐8 inhibitors that suppress the apoptotic execution cascade and mitigate endothelial injury. Pyroptosis can be modulated by agents such as colchicine or MCC950, which inhibit NLRP3 inflammasome assembly, or by VX‐765, which suppresses caspase‐1 activation, thereby limiting inflammatory cytokine release and endothelial pyroptosis. Ferroptosis can be targeted using iron chelators such as deferoxamine or by agents like ferrostatin‐1 that enhance GPX4 activity, reducing lipid peroxidation, and protecting endothelial cells.

Although the precise roles and interactions of PCD pathways in KD remain incompletely understood, therapeutic strategies targeting PCD‐related mechanisms offer promising directions for the development of novel treatments. Future research should focus on elucidating causal relationships between PCD and KD pathogenesis, validating the efficacy and safety of these interventions through multicenter clinical trials, optimizing therapeutic regimens, and ultimately improving outcomes for patients with KD, particularly those with concomitant coronary artery disease.

## Author Contributions

Bo Ding, Ziyu Huang, Ailixiati Alifu, Kaijing Wang, and Dufei Zhang wrote different sections of the manuscript. Yazhou Wang and Renwei Chen prepared, revised, and wrote the manuscript.

## Funding

This work was supported by the Hainan Province Clinical Medical Center (Grant QWYH202175).

## Disclosure

All authors listed have made a substantial, direct, and intellectual contribution to the work, and have read and approved the final manuscript for publication.

## Conflicts of Interest

The authors declare no conflicts of interest.

## Data Availability

All data presented in this review are from previously published studies and are cited accordingly; no new data were generated.
